# Supplementary material for: Analyzing the GHSI puzzle of whether highly developed countries fared worse in COVID-19
Source: Sci Rep. 2022 Oct 21;12:17711. doi: 10.1038/s41598-022-22578-2 (PMC9587258; doi:10.1038/s41598-022-22578-2)
Supplement: Supplementary file 1 — Supplementary Information. [file 41598_2022_22578_MOESM1_ESM.pdf]

Analyzing the GHSI puzzle of whether highly developed countries fared worse in COVID-19

Sofija Markovic[1], Igor Salom[2], Andjela Rodic[1], Marko Djordjevic[1,*]

[1]Quantitative Biology Group, Faculty of Biology, University of Belgrade, Serbia

[2]Institute of Physics Belgrade, National Institute of the Republic of Serbia, University of Belgrade, Serbia

1 Supplementary Figures

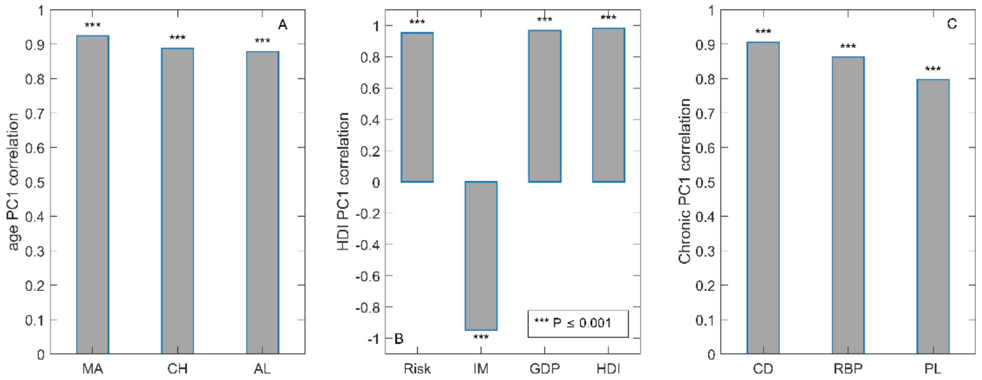

Supplementary Figure 1. Correlations of relevant principal components with variables entering PCA for m/r dataset. MA – median age, CH – blood cholesterol level, AL – alcohol consumption, Risk – GHSI Risk category, IM – infant mortality, GDP – gross domestic product per capita, HDI – human development index, CD – prevalence and severity of chronic diseases, RBP – prevalence of raised blood pressure, PL – long-term $PM_{2.5}$ pollution.

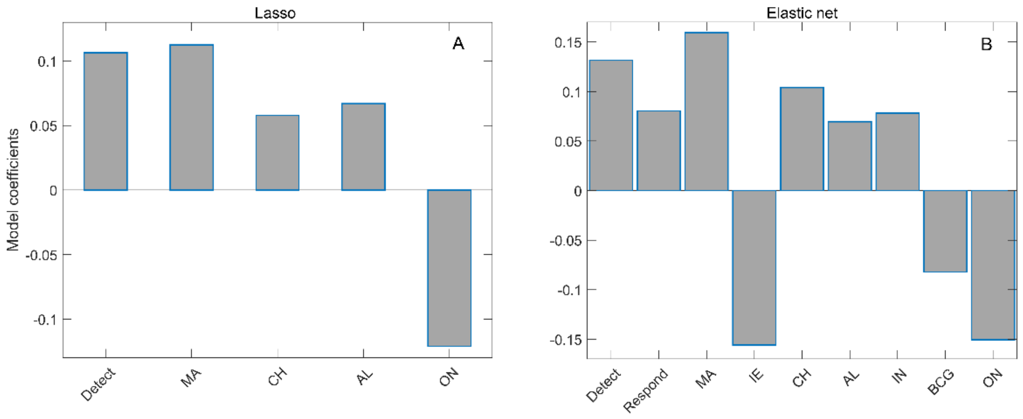

Supplementary Figure 2. Relaxed Lasso (A) and Relaxed elastic net (B) regressions on the initial set of demographic variables and Detect, Respond, Health and Risk GHSI categories. Detect – GHSI Detect category, Respond – GHSI Respond category, MA – median age, IE – net immigration, CH –

blood cholesterol level, AL – alcohol consumption, IN – prevalence of physical inactivity, BCG – BCG immunization coverage, ON – epidemic onset.

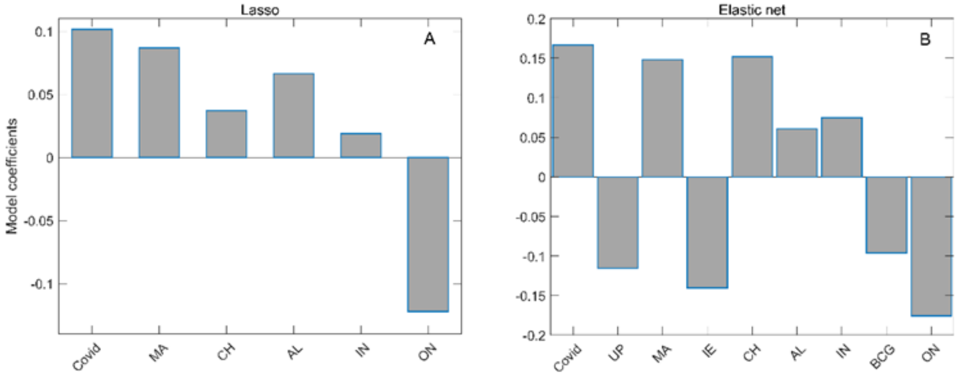

Supplementary Figure 1. LASSO (A) and Elastic net (B) on the initial set of demographic variables and Covid index. Covid – GHSI Covid category, UP – urban population, MA – median age, IE – net immigration, CH – blood cholesterol level, AL – alcohol consumption, IN – prevalence of physical inactivity, BCG – BCG immunization coverage, ON – epidemic onset.

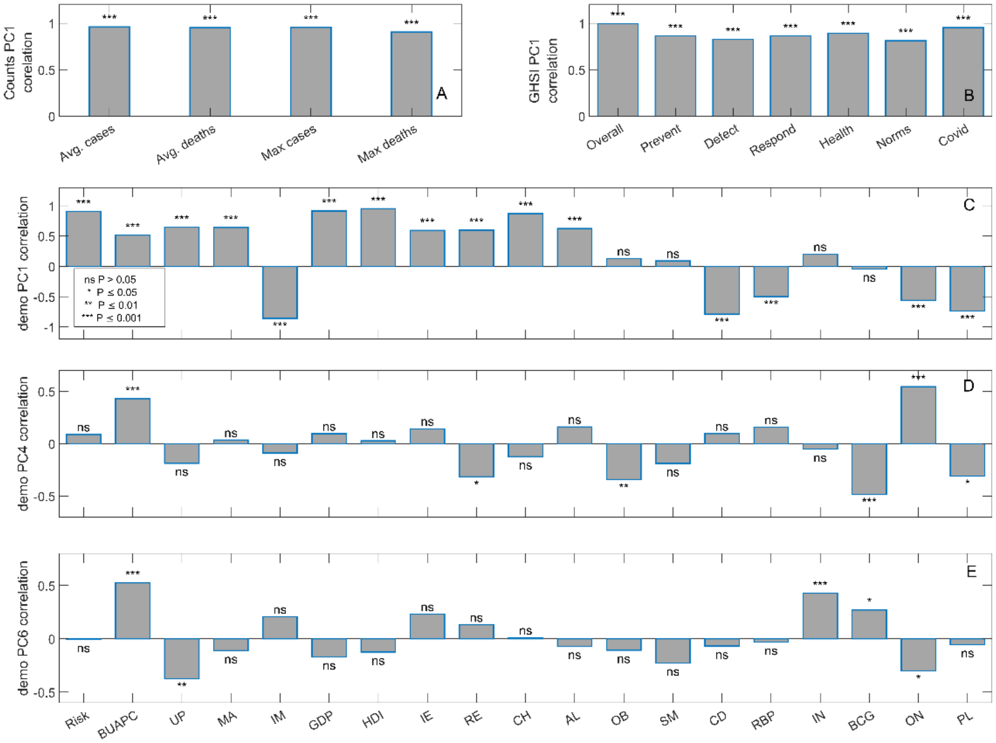

Supplementary Figure 2. Correlation of relevant principal components with the variables entering PCA in excess deaths dataset. Variable interpretation: A) Avg. cases- average daily COVID-19 cases, Avg. deaths - average daily COVID-19 deaths, Max cases- maximal daily number of cases, Max

deaths – maximal daily COVID-19 deaths B) GHSI indices C-E) Risk – GHSI Risk index, BUAPC – built-up area per capita, UP – urban population, MA – median age, IM – infant mortality, GDP – gross domestic product per capita, HDI – human development index, IE – net migration, RE – refugees, CH – blood cholesterol level, AL – alcohol consumption, OB – prevalence of obesity, SM – prevalence of smoking, CD – prevalence of cardiovascular diseases, RBP – raised blood pressure, IN – physical inactivity, BCG – BCG vaccination coverage, ON – the onset of the epidemic, PL – air pollution.

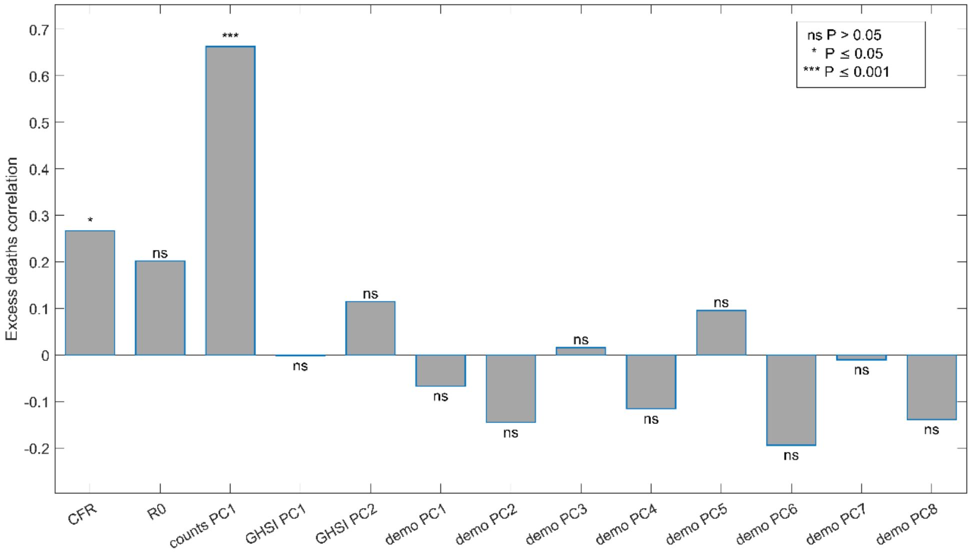

Supplementary Figure 3. Pearson's Correlation of excess deaths and selected variables and principal components. CFR – case fatality rate, R0 – basic reproduction number of the virus. For principal components explanation, see Supplementary Table 3.

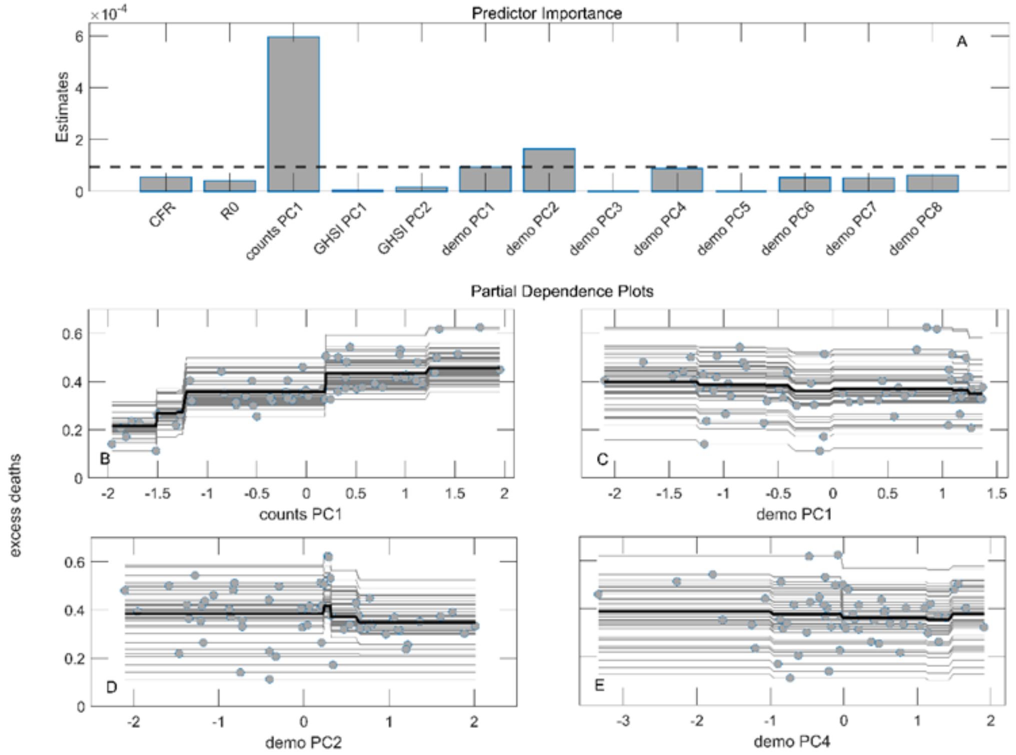

Supplementary Figure 6. Gradient Boost regression with excess deaths as the response variable. A) Predictor importance estimates. The dashed line represents the mean value of predictor importance. CFR – case fatality rate, R0 – basic reproduction number of the virus. For principal components explanation, see Supplementary Table 3. B-E) Partial dependency plots for selected variables with the highest importance estimates.

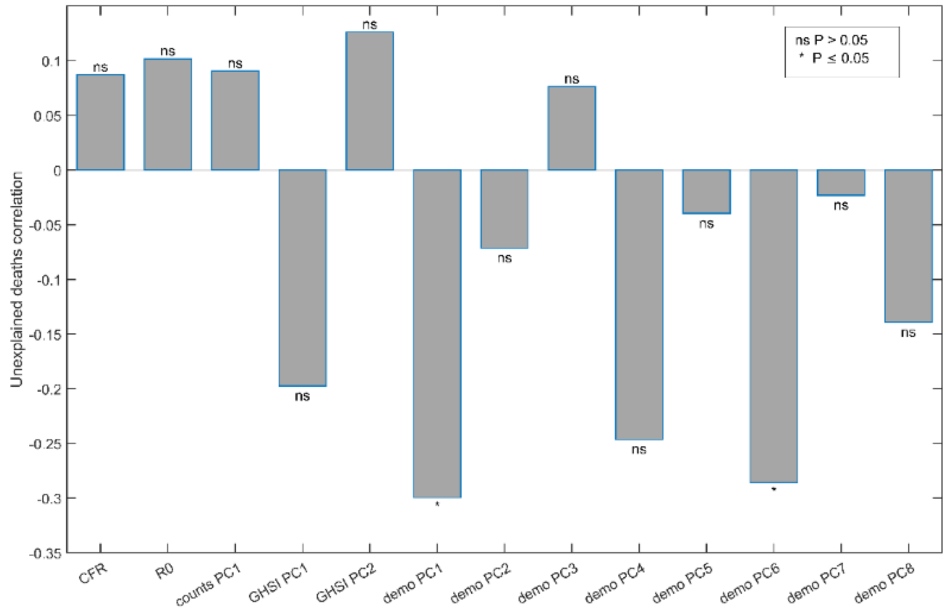

Supplementary Figure 4. Pearson's Correlation of unexplained deaths and selected variables and principal components. CFR – case fatality rate, R0 – basic reproduction number of the virus. For principal components explanation, see Supplementary Table 3.

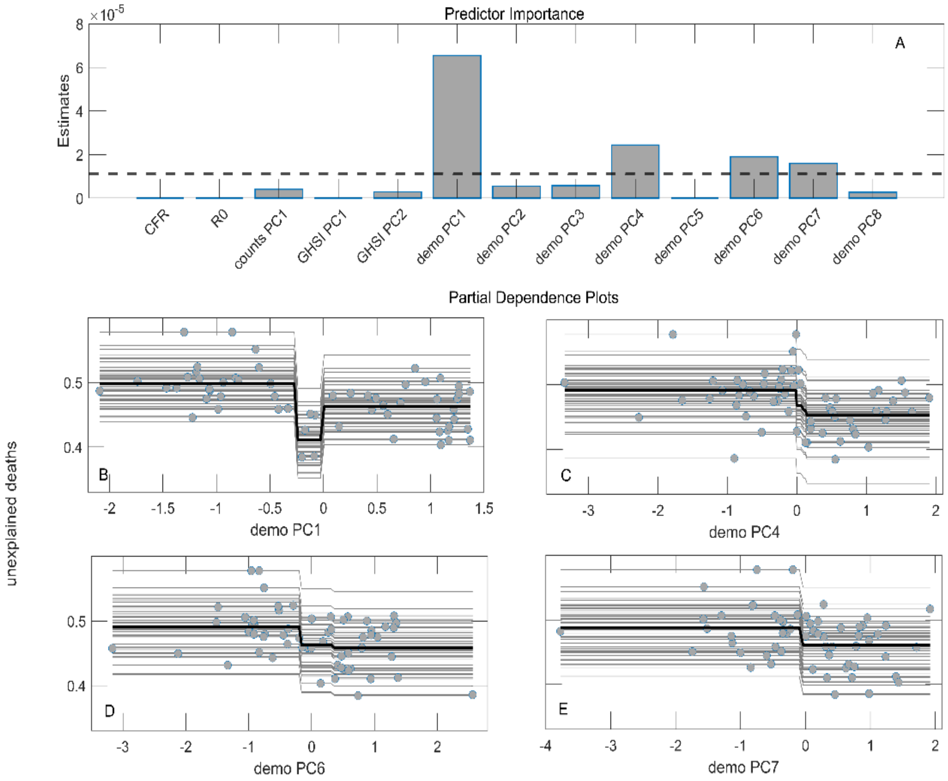

Supplement Figure 5. Gradient Boost regression with unexplained deaths as the response variable. A) Predictor importance estimates. The dashed line represents the mean value of predictor importance. CFR – case fatality rate, R0 – basic reproduction number of the virus. For principal components explanation, see Supplementary Table 3. B-E) Partial dependency plots for selected variables with the highest importance estimates.

2 Supplementary Tables

Supplementary Table 1: Data transformation

Variable	Abbreviation	Transformation in m/r dataset	Transformation in excess deaths dataset
Disease severity measure	m/r	$\log(x)$	/
Relative excess deaths	Excess deaths	/	$\sqrt{x - \min(x)}$
Relative unexplained deaths	Unexplained deaths	/	$\sqrt[3]{x - \min(x)}$
Case fatality rate	CFR	/	$\log(x)$
Basic reproduction number of SARS-CoV-2	R_0	/	$\log(x)$
Average daily cases	Avg. cases	/	$\log(x)$
Average daily deaths	Avg. deaths	/	$\log(x)$

Max daily cases (per capita)	Max cases	/	$\log(x)$
Max daily cases (per capita)	Max deaths	/	$\log(x)$
Overall GHSI	Overall	$\sqrt[3]{x}$	$\sqrt[3]{x}$
Prevent GHSI category	Prevent	None	None
Detect GHSI category	Detect	None	None
Respond GHSI category	Respond	$\sqrt[3]{x}$	$\sqrt[3]{x}$
Health GHSI category	Health	None	None
Norms GHSI category	Norms	None	None
Risk GHSI category	Risk	x^2	None
COVID-19 index	Covid	$\sqrt{x}$	$\sqrt{x}$
Built-up area per capita	BUAPC	$\sqrt{x}$	$\log(x)$
Urban population	UP	x^2	x^2
Median age	MA	None	$-\log(\max(x) - x)$
Infant mortality	IM	$\log(x)$	$\log(x)$
Gross domestic product per capita	GDP	$\log(x)$	$\log(x)$
Human development index	HDI	$-\sqrt{\max(x) - x}$	x^2
Net immigration	IE	$-\sqrt{\max(x) - x}$	None
Percentage of refugees	RE	$\log(x)$	$\log(x)$
Average blood cholesterol level	CH	$-\sqrt{\max(x) - x}$	x^2
Alcohol consumption	AL	None	x^2
Prevalence of obesity	OB	$-\log(\max(x) - x)$	$\sqrt[3]{x}$
Prevalence of smoking	SM	None	None
Prevalence and severity of chronic diseases	CD	$\sqrt[3]{x}$	$\log(x)$
Prevalence of raised blood pressure	RBP	$\sqrt{x}$	$\log(x)$
Prevalence of insufficient physical activity	IN	$-\sqrt{\max(x) - x}$	None
BCG immunization coverage	BCG	$-\sqrt{\max(x) - x}$	$-\sqrt{\max(x) - x}$
Epidemic onset (days from 15.02.2020. until the beginning of the epidemic in the given country)	ON	$\log(x)$	None
Long-term average $PM_{2.5}$ pollution	PL	$\log(x)$	$\log(x)$

“ / ” - indicates that variable was not included in the dataset, “None” - no transformation was applied.

Supplementary Table 2: Grouping of variables and PCA for m/r dataset

Variables entering PCA	Retained principal components	Variance explained
Risk	HDI PC1	92.7%

Infant Mortality<br>GDP per capita<br>Human development index		
Median age<br>Blood cholesterol level<br>Alcohol consumption	Age PC1<br>Age PC2	92.5%
Chronic diseases<br>Raised blood pressure<br>Pollution	Chr PC1<br>Chr PC2	91.0%

HDI PC1 – Human development index principal component 1, Chr PC1,2 – Chronic diseases principal components

Supplementary Table 3: Grouping of variables and PCA for excess deaths dataset

Variables entering PCA	Retained principal components	Variance explained
Average Daily Cases<br>Average Daily deaths<br>Max daily cases (per capita)<br>Max daily cases (per capita)	Counts PC1	89.0%
Overall GHSI<br>Prevent<br>Detect<br>Respond<br>Health<br>Norms<br>Covid	GHSI PC1<br>GHSI PC2	85.6%
Built-up area per capita<br>Urban population<br>Median age<br>Infant mortality<br>GDP per capita<br>Human development index<br>Immigrants – Emigrants<br>Refugees<br>Blood cholesterol level<br>Alcohol Consumption<br>Obesity<br>Smoking<br>Chronic diseases<br>Raised blood pressure<br>Physical inactivity<br>BCG vaccination coverage<br>Epidemic onset<br>Air pollution<br>Risk	Demo PC1<br>Demo PC2<br>Demo PC3<br>Demo PC4<br>Demo PC5<br>Demo PC6<br>Demo PC7<br>Demo PC8	86.8%

Supplementary Table 4: Excess deaths linear regression models

Stepwise linear regression				
Predictor	Estimate	SE	tStat	pValue
Counts PC1	0.10	0.015	6.7	10^{-8}
$R^2 = 0.44$, Adjusted $R^2 = 0.43$<br>P-value = 10^{-8}				

Lasso regression	
Predictor	Estimate
Counts PC1	0.072
$\lambda = 0.03$<br>min MSE = 0.74, SE min MSE = 0.032<br>$R^2 = 0.40$	

Elastic Net regression	
Predictor	Estimate
Counts PC1	0.073
$\alpha = 0.73$<br>$\lambda = 0.04$<br>min MSE = 0.72, SE min MSE = 0.036<br>$R^2 = 0.41$	

Supplementary Table 5: Unexplained deaths linear regression models

Stepwise linear regression				
Predictor	Estimate	SE	tStat	pValue
R_0	0.028	0.013	2.2	0.03
Demo PC1	-0.039	0.012	-3.1	0.003
Demo PC6	-0.033	0.012	-2.8	0.007

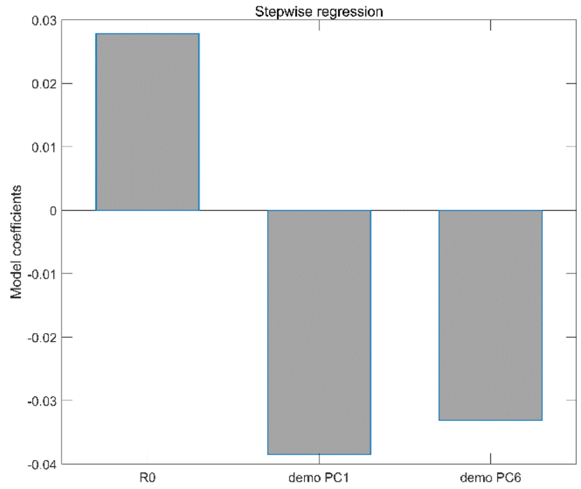

$R^2 = 0.24$, Adjusted $R^2 = 0.20$
P-value = 0.002

Lasso regression	
Predictor	Estimate
Demo PC1	-0.009
Demo PC4	-0.004
Demo PC6	-0.008

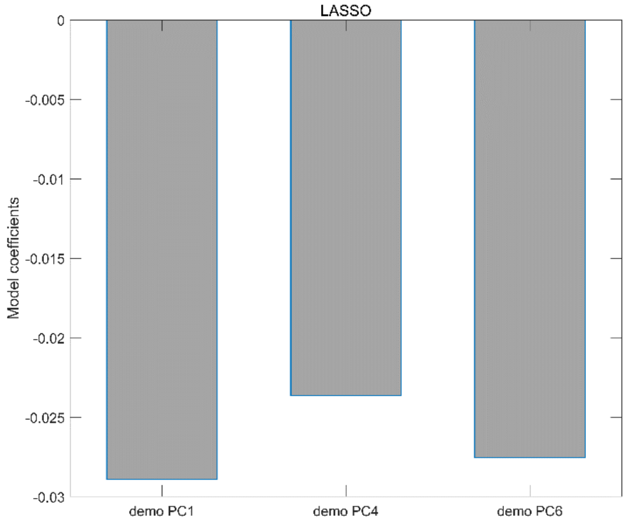

$\lambda = 0.02$
min MSE = 1.1 , SE min MSE = 0.030
$R^2 = 0.10$

Elastic net regression	
Predictor	Estimate
Demo PC1	-0.011
Demo PC4	-0.0058
Demo PC6	-0.0097

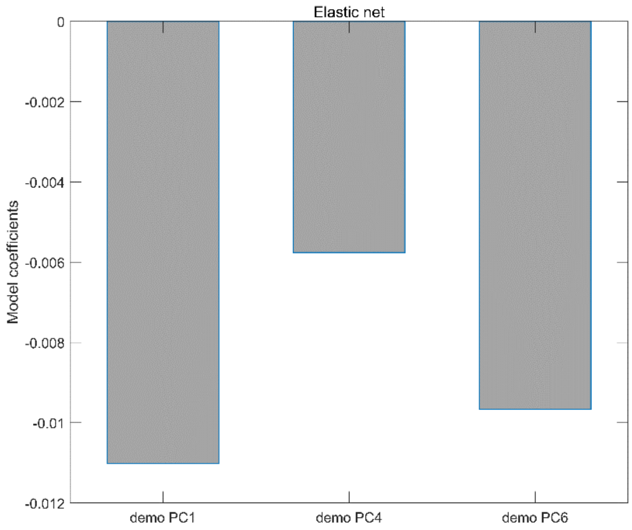

α = 0.83<br>λ = 0.02<br>min MSE = 1.12, SE min MSE = 0.030<br>R^2 = 0.13
